# Presenting an Ideal Self on Weibo: The Effects of Narcissism and Self-Presentation Valence on Uses and Gratifications

**DOI:** 10.3389/fpsyg.2020.01310

**Published:** 2020-06-11

**Authors:** Lei Vincent Huang, Susu Liu

**Affiliations:** ^1^Department of Communication Studies, School of Communication, Hong Kong Baptist University, Kowloon, Hong Kong; ^2^School of Social Sciences, Soochow University, Suzhou, China

**Keywords:** narcissism, self-presentation valence, uses and gratifications, social media, Weibo, mediation

## Abstract

Narcissism has been shown to have a positive and significant impact on uses of microblog services and features for self-presentation (SP). Little is known with regard to whether and how narcissism and valence of online SP could jointly influence the gratifications of microblog uses. This study explores how narcissism influences the uses and gratifications of Weibo via positive and negative SP. Results from a survey of Weibo users in China suggest that narcissism generates positive effect on both gratification sought (GS) and gratification obtained (GO). GS fully mediates the effect of narcissism on GO. Moreover, positive SP plays a mediating role between GS and GO, while negative SP does not. The mediation mechanisms within narcissism, the valence of SP, GS, and GO suggest that differentiating the gratification processes could help us better understand uses and gratification of microblog services.

## Introduction

Communication technologies such as social network service (SNS) offer unprecedented opportunities for users to manage and control their online self-presentation (SP). Users post on SNSs for various reasons, such as constructing desired identities, gaining social influence, and seeking for social validation (Sundar and Limperos, 2013). These activities can be more strategic and be much more controlled, as compared to traditional face-to-face interactions (Walther, 2007; Bibby, 2008; Krämer and Winter, 2008). Online SP is one of the mostly studied topics in cyberpsychology scholarship (Toma et al., 2008; Mehdizadeh, 2010; Grieve and Watkinson, 2016; Twomey and O’Reilly, 2017). Many studies have explored how personalities could affect how people present themselves online, most of which are on the relationship between narcissism and SP (Buffardi and Campbell, 2008; Mehdizadeh, 2010; Ong et al., 2011). Although previous studies have shown that narcissism has a significant impact on online SP, scholars have yet to explore how this personality trait could influence valence of SP. Filling this gap, this study investigates the relationship between narcissism and valence of online SP from the lens of uses and gratification (U&G) theory (Katz et al., 1973, 1974).

### Narcissism, Self-Presentation, and SNS Use

Narcissism is defined as a highly inflated, positive but unrealistic self-conception, a lack of interest in forming strong interpersonal relationships, and a strategic self-regulatory engagement affirming the positive self-views (Campbell and Foster, 2007; Nardis and Panek, 2019). SNSs provide excellent platforms for narcissists, because SNSs allow almost full control over different types of SP. Meanwhile its anonymous and rapid spreading feature increase the positivity of their self-conceptions based on superficial relationships (Buffardi and Campbell, 2008). In this regard, narcissistic users are prone to take advantage of SNSs to construct self-images to obtain more attractiveness. Scholars have also identified that those high on narcissism and low on self-esteem are more likely to maintain a positive SP on Facebook or Twitter (Mehdizadeh, 2010).

As a typical SNS that provides affordances of content editability and open communication, Weibo coincides with a certain group of people with narcissistic personalities. The ubiquitous nature of microblogging provides a fertile ground for users to share updates and photos with a large number of people anytime and anywhere (Mehdizadeh, 2010; Mo and Leung, 2015). Various types of SP, characterized as SP valence, can be detected on Weibo platform, including positive photo exhibition, nude or ugly self-disclosure and even unfavorable information (White et al., 2018). Valence in psychology is used to discuss the “good”-ness (positive valence) or “bad”-ness (negative valence) of an event, object, or situation (Frijda, 1986). In general, SNS users are more likely to post positive contents (e.g., beautified photos, successful or inspirational experiences, delightful events, etc.) for the sake of portraying a positive image of oneself (Gibbs et al., 2006; Hancock et al., 2007; Ellison et al., 2012). Nevertheless, a large amount of negative contents appear in Weibo and this trend has witnessed a dramatic increase in recent years. The negative valence of SP consists of nude or ugly selfies, bad-looking photos, unpleasant emotions, and potentially damaging information (White et al., 2018). These behaviors seem to run counter the tenets of exhibiting an ideal self on Weibo. It is debated that negative SP might be driven by individuals not spending time and cognitive efforts on thinking about the potential negative effects (White et al., 2018). Others indicate that negative SP is associated with an inclination to gratify desires for the purposes of enjoyment or the gratification to attract social attention (Hofstede, 2001; White et al., 2018). Hence, it is vital to understand the processes that might underlie the propensity to display both positive and negative types of contents on SNSs.

Since narcissistic users would pay more attention to their SP, it seems that narcissism would influence valence of online SP. However, to the best of our knowledge, no existing empirical research has explored the impact of narcissism on SP valence. Filling this research gap, this study examines the effects of narcissism and SP valence on Weibo use from the perspective of U&G theory.

### Uses and Gratification, Narcissism, and SNS Use

Recognized as a “sub-tradition” of media effect research, U&G theory focuses on what people do with media, shifting away from traditional media effect studies querying “what media do to people” to “what people do with media” (Katz, 1959; McQuail, 2004). Adopting a psychological perspective to study media audience, U&G theory argues that media users are active in being aware of their social and psychological needs and then become goal-orientated and gratification-expected in media use. Media users access to the media and then get some or no gratification which would affect their decision next time. U&G theory explains that media users hold expectations before they choose the media and take actions. The activity with media is driven by the sought of gratification and then affects the gratification obtained (GO) from the experience with media (Katz et al., 1973; Palmgreen and Rayburn, 1979). Gratification sought (GS) connotes expectations users have in terms of gratifications that could be obtained from media use, which motivates media use. GO is an outcome, which refers to experience of media use or actual fulfillment after media use.

### Hypothesis of the Study

SNS users are offered many opportunities for strategic SP. A body of prior research has discovered the associations between personality traits and social media usage. Personality traits in the context of “Big-five” model (e.g., extraversion, neuroticism, and openness), self-esteem, and narcissism have been unraveled to affect the motivation or gratification desire in SNS use (Correa et al., 2010; Ong et al., 2011; Zhang et al., 2011). In accordance with these findings, Buffardi and Campbell (2008) confirmed that narcissism predicted higher levels of online social interactions and more self-promoting content in social media. Parallel to this line of thought, this study proposes the following hypotheses:

H1a: Individuals with higher narcissism scores will be associated with a larger amount of Gratification Sought.
H1b: Individuals with higher narcissism scores will be associated with a larger amount of Gratification Obtained.

Several mechanisms inherent in Weibo may benefit users with different social psychological characteristics, such as narcissism. The editability of Weibo allows users to create and change self-relevant information before they transmit their messages because they have enough time to deliver satisfactory posts (Mo and Leung, 2015). Narcissistic users can take full use of various image editing apps and compose texts meticulously before sending out. Photos can be constructed and refined to conceal flaws, which appears positive and attractive. Personal experiences can be boasted or exaggerated as successful and fascinating as an approach of self-promoting. These actions belong to positive valence of SP, enabling narcissists to maintain positive online personas. Moreover, negative valence of SP on Weibo is also concerned in the present study to further explore how different negative posts are related to narcissistic users. Presenting a negative online persona may not necessarily be the norm, with many considering this type of behavior idiosyncratic. However, negative SP such as posting bad-looking photos, discouraging emotions, and negative side of personality can be found among Weibo users. Previous research suggests that people who habitually emphasize negative things about themselves might result from releasing bad emotions or pressures (White et al., 2018). Nevertheless, this negative SP is considered to deviate from the purposes that narcissists seek to realize. Cantor and Norem (1989) posited that negative disclosure may alienate individuals’ social support networks, which, to a large extent, hinders narcissists in constructing an ideal self.

It should be noted that we regard positive and negative SP as independent valence measures. Indeed, a narcissist may be motivated to engage in more positive and less negative SP, which indicates that positivity and negativity are two extremes of a single continuum. However, people may also present both positive and negative selves in their posts if their motivation is not to present ideal self-images. Previous studies (e.g., Hollenbaugh and Ferris, 2015; Gibbs et al., 2006) usually only measure amounts of positive SP, which overlook how people control negative SP. To overcome this issue, in the present study, we measure positive and negative SP separately. Building on the existing literature on narcissism and SP valence, this study proposes the following hypotheses:

H2a: Individuals with higher narcissism scores will be associated with a larger amount of positive self-presentation.
H2b: Individuals with higher narcissism scores will be associated with a smaller amount of negative self-presentation.

Grounded in the tenets of U&G theory and the relationships specified in previous hypotheses, those with high narcissism scores are supposed to trigger the gratification desire and the expectations of “hoped” gratification by means of SP valence (Seidman, 2013). Personality traits may serve as antecedents that reinforce motivations related to SNS usage and ultimately trigger the intensity of usage (Ross et al., 2009). Thereby, in an effort to obtain gratification through Weibo usage, SP and GS behaviors might serve as mediating roles in the process. In case of the current research, another set of hypotheses are proposed as follows:

H3a: Gratification Sought will mediate the effect of narcissism on Gratification Obtained.
H3b: Self-presentation valence will mediate the effect of Gratification Sought on Gratification Obtained.

## Materials and Methods

### Participants and Procedure

To test the proposed hypotheses and research questions, an online survey was conducted on Weibo using the snowball sampling method (questionnaire items are available in Supplementary Material). A private message including an invitation and a link generated by an online survey company was sent to Weibo users. A number of 306 users responded to the invitation and finished the questionnaire. Extracted from the survey company, all the data were input to IBM SPSS 21.0 and AMOS 21.0 for analysis. Bivariate correlation analysis, hierarchical regression analysis and path analysis were adopted to verify the hypotheses, respectively.

### Measures

#### Narcissism

This study adopts the measurement in Asada et al.’s (2004) study to measure respondents’ narcissism. The reliability of the scale is highly satisfactory (α = 0.83). This measurement is an 8-item, 7-point Likert-scale (ranging from 1-not at all to 7-very much) basing on the conceptualization that “narcissism is the tendency to inflate one’s fragile self-image at the cost of others and narcissist people have extreme sense of grandiosity and exhibitionism” (p. 383). The mean score of the eight items were calculated as the scale’s value.

#### Valence of Self-Presentation

Self-presentation is divided into positive and negative valence, which is measured in a 10-item, 7-point Likert-scale (ranging from 1-not at all to 7-very much). The questionnaire uses scales adjusted from Park et al. (2009). To validate the discriminative validity of the scale of SP valence, factor analysis was conducted. After deleting two items of negative SP in the original scale, an analysis of the Kaiser–Meyer–Olkin (KMO) measure of sampling adequacy suggested that the sample was factorable (KMO = 0.682). The result of the Bartlett’s test of sphericity was significant (*p* < 0.01). The results of the principal component factor analysis yielded a two-factor solution that explained 52.53% of the total variance. Hence, the results supported an eight-item scale of SP valence. Additionally, the Cronbach’s α of the SP scale is 0.73 for the positive valence and 0.50 for the negative valence. It should be noticed that the reliability of negative SP is below the minimum acceptable limit (Cronbach’s α = 0.6; Dai, 2015). However, in the present study we could moderately accept it because the scale of negative SP only consisted of two items. The mean score of the items for positive and negative SP were calculated, respectively.

#### Gratification Sought

Items of self-expression related GS in previous studies (Papacharissi and Rubin, 2000; Quan-Haase and Young, 2010) were adopted and revised to measure the expectation of Weibo use. A 6-item, 7-point Likert-scale (ranging from 1-not at all to 7-very much) is used. The reliability of the scale is acceptable with a Cronbach’s α of 0.77. The mean score of the six items were calculated as the scale’s value.

#### Gratification Obtained

A four-item 7-point Likert-scale (ranging from 1-not at all to 7-very much) is adopted to study the construct of GO. The Likert-scale adopted and revised from a previous communication satisfaction inventory (Hecht, 1978) measures the extent of gratification for usage of Weibo in terms of expressing ideal self. The Cronbach’s α of the scale of GO is satisfactory (α = 0.84). The mean score of the four items were calculated as the scale’s value, such that higher scores indicating higher GO.

## Results

As it was set from the online survey company that only the respondent who filled in all the questions could submit the results to database, all questionnaires in record were valid. Among 306 participants, 164 (53.6%) were male, 138 (45.1%) were female, and 4 (1.3%) chose not to indicate the gender. In terms of age, 15 (4.9%) of them are between the range 16–20, 252 (82.4%) are 21–25, 36 (11.8%) are 26–30 and 3 (1.0%) are 31 or above.

A series of Pearson correlation analysis were conducted to test the relationships between variables proposed in hypotheses (see Table 1). Narcissism scale was positively correlated with GS (*r* = 0.334, *p* < 0.001) and positively correlated with GO (*r* = 0.279, *p* < 0.001). Narcissism was also found to be positively correlated with positive SP (*r* = 0.457, *p* < 0.001) and negatively correlated with negative SP (*r* = −0.133, *p* < 0.001). Therefore, Hypothesis 1a,b, together with Hypothesis 2a,b were supported.

**TABLE 1:** Descriptive statistics for and zero-order correlations among study variables in the model.

	M (SD)	2	3	4	5
1. Narcissism	4.07 (0.91)	0.457***	−0.133*	0.334***	0.279***
2. Positive SP	4.34 (0.87)		−0.297***	0.515***	0.447***
3. Negative SP	4.16 (1.14)			−0.096	0.045
4. GS	4.78 (0.85)				0.419***
5. GO	4.77 (0.99)				

*p < 0.05, ***p < 0.001. SP, self-presentation; GS, gratification sought; GO, gratification obtained.

To further examine the effects of narcissism and SP valence on Uses and Gratifications, hierarchical linear regression analysis was adopted and the results were exhibited in Table 2. In addition to the significant associations identified in correlational analysis, regression analysis showed that both narcissism (β = 0.151, *p* < 0.001) and GS (β = 0.376, *p* < 0.001) generated a positive effect on GO. So GS mediated the effect of narcissism on GO and H3a was preliminary supported.

**TABLE 2:** Regression coefficients of self-presentation, gratification sought and gratification obtained on main variables (standardized coefficients are shown, *N* = 306).

Variables	GS	GS	GO	GO	Positive SP	Negative SP	GO
	
	Model 1	Model 2	Model 3	Model 4	Model 5	Model 6	Model 7
Age (21–25 years)							
16–20 years	0.203***	0.219***	0.060	–0.023	0.001	0.082	–0.050
26 and above	–0.035	–0.041	0.045	0.061	0.092	–0.084	0.047
Gender (Male)							
Female	–0.028	–0.027	–0.031	–0.021	–0.028	–0.034	–0.004
Narcissism		0.345***	0.281***	0.151**			0.057
Positive SP							0.251***
Negative SP							0.349***
GS				0.376***	0.518***	−0.118*	0.187***
*R*^2^	0.044	0.163	0.084	0.202	0.275	0.024	0.285
*R*^2^ change		0.119		0.118			0.083

*p < 0.05, **p < 0.01, ***p < 0.001. SP, self-presentation; GS, gratification sought; GO, gratification obtained.

**FIGURE 1:**
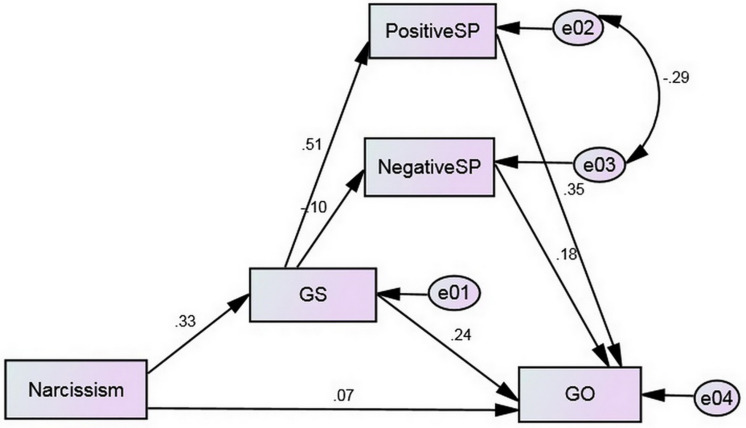
Results of multi-mediation analysis for GO. All the coefficients were significant standardized values. SP, self-presentation; GS, gratification sought; GO, gratification obtained. All the coefficients were significant standardized values *(p <* 0.001), except for the path from narcissism to GO (β = 0.07, *p* = 0.209) and the path from GS to negative SP (β = –0.10, *p* = 0.091).

Gratification sought was positively associated with positive SP (β = 0.518, *p* < 0.001) and negatively associated with negative SP (β = −0.118, *p* < 0.05), respectively. Moreover, positive SP, negative SP and GS have shown a positive effect on GO, respectively in Model 7. H3b was preliminary supported. Furthermore, path analysis was employed to assess the multiple mediation effect. The influences of the demographic variables (i.e., gender and age) were entered as covariates during the analysis. The statistics demonstrated two joint mediation models of key variables, as Figure 1 showed. The mediation effect of GS on narcissism and GO was confirmed. GS fully mediated the effect of narcissism on GO. In addition, the mediation effect of positive SP on GS and GO was also demonstrated. However, statistics could not support the mediating role of negative SP on GO. Furthermore, Sobel test was conducted to test the significance of indirect effect of narcissism on GO through GS (β = 0.133). The result showed that the indirect effect was significant (*Z* = 3.33, *p* < 0.001). Therefore, H3a was supported. Sobel test also showed that the indirect effect of GS on GO through positive SP was significant (β = 0.164, *Z* = 5.17, *p* < 0.001). Thus, H3b was partially supported.

## Discussion

This article aims at exploring the relationships among narcissism, valence of SP, GS and obtained from Weibo uses. As suggested by the results, users of Weibo who are more narcissistic engage in a higher level of positive SP and a lower level of negative SP. However, only the former one has a significant impact on GO.

One major contribution of this article is to bring in valence of SP to examine how different kinds of SP influence uses and gratification of microblogging service. Prior research has been focusing on how narcissism, as an offline personality trait, could influence online behaviors, especially uses of SNS function for SP (Mehdizadeh, 2010; Ong et al., 2011; Mo and Leung, 2015). Little is known, however, with regard to whether and how narcissism influences content construction on SNS. As an initial attempt to fill this research gap, this study introduces valence of SP. Consistent with existing studies, we find that narcissism has a positive impact on using microblog to obtain gratification and yet we go a step further to show that the positive impact of narcissism on GO was only through GS and positive SP.

Another contribution of this study is that we linked GS and GO and explored how this relationship is affected by narcissism and SP activities. Findings suggest that those who are more narcissistic are more likely to seek gratification opportunities. Research has shown that people would seek different gratifications from using social media, such as multifunctionality and synchronicity (Lo and Leung, 2009). Future research could draw on existing research to explore how narcissism influences different kinds of gratifications users seek from using social media. It will also be interesting to compare how narcissistic users seek different gratifications from different kinds of social media. For instance, narcissistic users may seek image-related gratification from SNS whereas opinion-related gratification from microblog services. Moreover, U&G research has differentiated gratification people seek and obtain and yet few studies later explored the relationship between GS and GO (Mo and Leung, 2015). It is understandable because new media scholars would be interested in motives of using new media and thus would focus more on GS. Nevertheless, it is of value to explore the relationship between GS and GO as it will lay the ground for exploration of processes that make social media use gratifying. In the context of narcissism and SP, we found that those who are more narcissistic are more likely to seek gratification and through positive SP, these users are more likely to feel gratified from using microblog. Future research can extend this study by exploring how narcissism is associated with different kinds of gratifications and how other communicative processes, such as social interactions, play a role in the mechanism between GS and GO.

## Limitations and Conclusion

Several limitations of this study should be noted. One limitation is that it draws on data collected through convenience sampling in a cross-sectional study, which may limit the generalizability of the findings. Most of the respondents in this study are emerging adults. Thus, findings of this study may only shed lights on users of this age group. Another limitation is that survey measures on SP valence only captures perceived SP valence. Future research could content analyze users’ posts with regard to their valence and investigate its relationship with narcissism and gratification measures.

This study extends previous research on narcissism and online SP to explore how valence of SP plays a role in uses and gratification of microblog services. It also uncovers how valence of SP mediates the relationship between GS and GO. Overall, this study shows that narcissistic users are more likely to seek gratification from using microblog services and that gratification is more likely to be obtained from positive, rather than negative, SP.

## Data Availability Statement

The datasets generated for this study are available on request to the corresponding author.

## Ethics Statement

Ethical review and approval were not required for the study on human participants in accordance with the local legislation and institutional requirements when the study was conducted. Informed consent was inferred through the completion of the survey.

## Author Contributions

LH contributed to the research design and data collection. SL contributed to the data analysis. LH and SL contributed to the hypotheses initiation and manuscript writing.

## Conflict of Interest

The authors declare that the research was conducted in the absence of any commercial or financial relationships that could be construed as a potential conflict of interest.
