# Supplementary material for: Presenting an Ideal Self on Weibo: The Effects of Narcissism and Self-Presentation Valence on Uses and Gratifications
Source: Front Psychol. 2020 Jun 11;11:1310. doi: 10.3389/fpsyg.2020.01310 (PMC7300319; doi:10.3389/fpsyg.2020.01310)
Supplement: Supplementary file 1 [file Table_1.DOCX]

**﻿Measurement items and sources**

﻿Note. All the items were measured on 7-point Likert scales, asking the extent to which the participants agreed on the statements, ranging from 1-not at all to 7-very much.

**Narcissism (adopted from Asada et al., 2011)**

1. Deep down I think I am better than most other people.
2. Other people often envy me.
3. I am better than most other people.
4. I enjoy being the center of attention.
5. It is important to me that others know about my success.
6. I sometimes "show off" to other people.
7. I like talking about myself
8. I like people to pay attention to me.

**Valence of Self-presentation (modified from Park et al., 2009)**

*Positive Self-presentation*

1. I will always ensure that the content about me on Weibo is positive.
2. I modify the content that I post to Weibo to make myself look better.
3. I like presenting positive things about myself on Weibo.
4. I like sharing my successful experience on Weibo.
5. I use apps or features of Weibo to retouch my photos before posting them.
6. If I am in a photo that is to be uploaded to Weibo, I must look good in the photo.

*Negative Self-presentation*

1. I do not mind posting or someone else posting pictures in which I look bad to Weibo.
2. I do not mind posting negative side about myself on Weibo.
3. I would rather not post a photo if it does not look good. (dropped)
4. I share my failure or upset experience on Weibo. (dropped)

**Gratification Sought (GS) (modified from Quan-Haase & Young, 2010; Papacharissi & Rubin, 2000)**

1. I expect to receive positive feedback on my Weibo posts.
2. I expect to receive negative feedback on my Weibo posts. (reverse coded)
3. I expect others to like or repost my photos.
4. I expect others to like or repost my videos.
5. I expect others to envy me for my interesting experience or successful stories shared on Weibo.
6. I expect to get more followers in microblog.

**Gratification Obtained (GO) 4 item (modified from Hecht,1978)**

1. Weibo is a good tool for me to express myself.
2. I feel satisfied that microblog helps me build a good image of myself.
3. I have received much attention from others through using Weibo.
4. I make people know me well through using Weibo.
